# Knockdown of TXNDC9 induces apoptosis and autophagy in glioma and mediates cell differentiation by p53 activation

**DOI:** 10.18632/aging.103915

**Published:** 2020-09-08

**Authors:** Tingting Zheng, Keke Chen, Xue Zhang, Huanhuan Feng, Yu Shi, Li Liu, Jun Zhang, Yun Chen

**Affiliations:** 1 Shenzhen Key Laboratory for Drug Addiction and Medication Safety, Department of Ultrasound, Peking University Shenzhen Hospital, Shenzhen Peking University, The Hong Kong University of Science and Technology Medical Center, Shenzhen, Guangdong Province, China; 2 Clinical College of Shenzhen Hospital, Peking University, Anhui Medical University, Shenzhen, Guangdong Province, China; 3 School of Materials Science and Engineering, Harbin Institute of Technology Shenzhen, Shenzhen, Guangdong Province, China; 4 Queensland Micro- and Nanotechnology Centre, Griffith University, Brisbane, Australia

**Keywords:** glioma, TXNDC9, apoptosis, autophagy, differentiation

## Abstract

Glioma is the most common malignant brain tumor. Because of its high degree of malignancy, the effect of surgical treatment, radiotherapy, chemotherapy, or immunotherapy is not ideal. TXNDC9 belongs to thioredoxin domain-containing proteins, which is involved in tumor progression. However, no research associated with TXNDC9 has been reported in glioma. In this study, we found that TXNDC9 was upregulated in glioma. Knockdown of TXNDC9 would prevent proliferation and metastasis, induce the apoptosis rate of glioma cells, and promote the expression Cleaved-caspase3, Cleaved-caspase8, Cleaved-caspase9. Meanwhile, knockdown of TXNDC9 induced autophagy by increasing the level of LC3 and Beclin-1. Cell morphology and expression analysis of GFAP, Vimentin, verified that TXNDC9 could regulate glioma cell differentiation. During this program, the expression of p53 changes dramatically. The apoptosis, autophagy, and cell differentiation program were blocked by p53 inhibitor treatment. In conclusion, the silencing of TXNDC9 induces apoptosis and autophagy in glioma and promotes cell differentiation by controlling p53 and may function as a new mechanism in glioma.

## INTRODUCTION

Glioma is the most frequent primary tumor in the brain [1]. It has the characteristics of high incidence, invasive growth, and recurrence [2, 3]. It has become a significant problem affecting human health. Therefore, it is essential to investigate the mechanism involved in the development and progression of glioma.

The proliferation of tumor cells is regulated by programmed cell death. Autophagy and apoptosis are two forms of programmed cell death [4]. They have significant differences in morphology and function, but there are also many connections, which are related to the activation, expression, and regulation of a series of genes. It was reported that Licarin A induces autophagy and apoptosis in NSCLC cells [5]. Allavena G et al. found that targeting translational machinery can be made for the elimination of autophagy-deficient cells through the CASP8-dependent apoptotic signal pathway in NSCLC cells [6]. W09 would promote autophagy-dependent cell apoptosis by regulating the Ras/MAPK signal pathway [7]. The silencing of cadherin-17 enhances apoptosis and inhibits autophagy in colorectal cancer cells [8]. With the deepening of research, more and more studies have proved that there is a specific relationship between apoptosis and autophagy. Whether autophagy can be regulated or not is closely related to the growth and apoptosis of tumor cells.

A malignant tumor is a common clinical disease, which seriously threatens people's life and quality of life. Since Pierce et al. first discovered that mouse testicular teratoma cells could spontaneously differentiate into normal cells in 1960, more and more studies have shown that dedifferentiated tumor cells can also be induced and re-differentiated into normal cells under the action of differentiation inducers, and their biological characteristics gradually move closer to normal cells and even transform into normal cells. It was reported that miR-146a/TRAF6 induced Th17 cell differentiation to control cervical cancer cell growth and apoptosis via NF-κB signaling [9]. NELL1 could regulate cell differentiation in lung cancer [10]. EGFR/AKT signaling pathway involved in ovarian cancer cell differentiation via regulating TSA [11].

Thioredoxin domain-containing 9 (TXNDC9) belongs to the TNX family. Recently research found that TXNDC9 benefited oxaliplatin resistance via regulation of autophagy and apoptosis in colorectal adenocarcinoma [12]. TXNDC9 also accelerated the development of prostate cancer via regulating oxidative stress-induced androgen receptor signaling [13]. TXNDC9 facilitated the program of hepatocellular carcinoma [14]. TXNDC9 might be a potential biomarkers in Alzheimer's disease diagnosis [15].

In this study, we stated that TXNDC9 would be a tumor-associated gene, which involved in the development of glioma. Knockdown of TXNDC9 could prevent tumor program, induce apoptosis, and autophagy in U87 and U251 cells. Moreover, TXNDC9 prompted differentiation in U87 and U251 cells through the p53 signal pathway.

## RESULTS

### TXNDC9 was up-regulated in glioma tissues and cells

**Figure 1:**
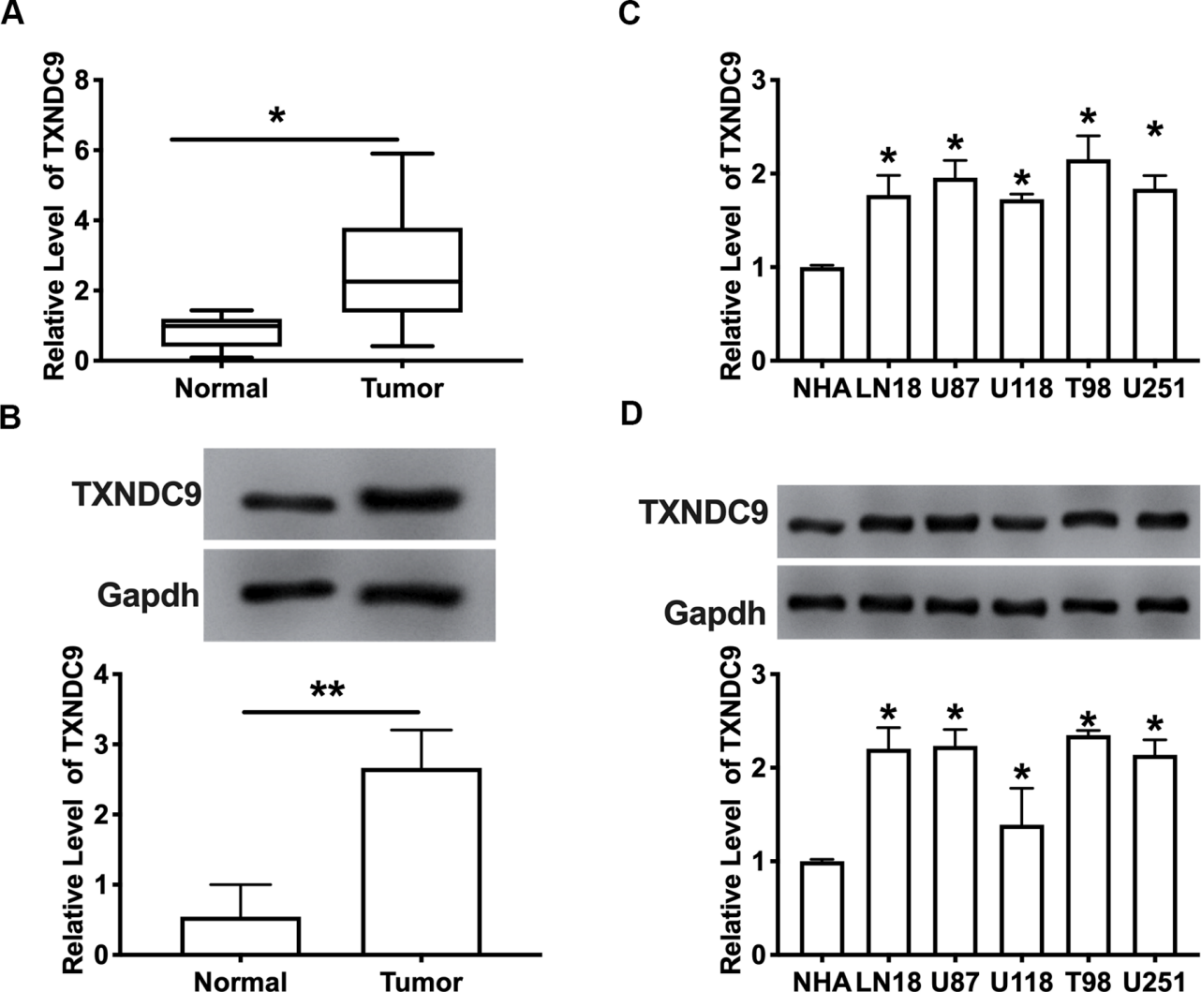
**TXNDC9 was upregulated in glioma tissues and cells.** (**A**) The mRNA level of TXNDC9 in tumor and normal samples was detected by RT-PCR. n=40, *P<0.05. (**B**) The protein level of TXNDC9 in tumor and normal samples was detected. n=6, *P<0.05. (**C**) The mRNA level of TXNDC9 in different glioma cell lines (LN18, U87, U118, T98, U251) were detected by RT-PCR. NHA cell was indicated as a control. n= 6, *P<0.05. (**D**) The protein level of TXNDC9 in different glioma cell lines. n= 4, *P<0.05.

To explore the role of TXNDC9 in glioma, we first detected the expression of TXNDC9 in glioma tissue and normal tissue. The results showed that the mRNA and protein expression level of TXNDC9 was an upregulation in tumors compared with normal tissues (Figure 1A, 1B). Then we measured the mRNA and protein level of TXNDC9 in different human glioma cell lines (LN18, U87, U118, T98, U251) and human astrocytes (NHA) was used as a control. Compared with NHA, the expression of TXNDC9 was up-regulated in all glioma cell lines (Figure 1C, 1D).

### Effects of TXNDC9 on cell metastasis and apoptosis in U87 and U251 cells

**Figure 2:**
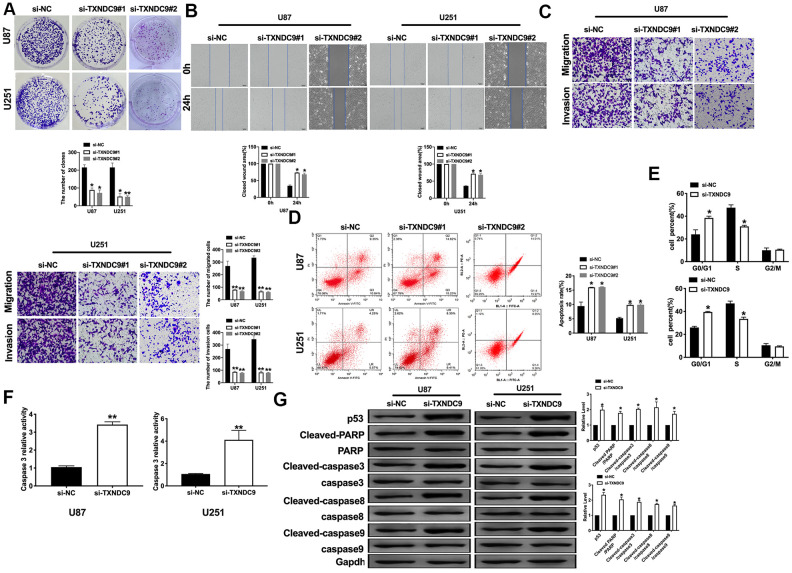
**Knockdown of TXNDC9 prevented proliferation and induced apoptosis of U87 cells.** (**A**) The colony formation assay. (**B**, **C**) Wound healing assay and transwell were performed for detecting the effect of TXNDC9 on migration and invasion. (**D**) The apoptosis rate of U87 and U251 cells were measured by flow cytometry. The histogram at the right is a statistical graph. n=4, *P<0.05. (**E**) Flow cytometry was performed to determine the cell cycle in U87 and U251 cells after transfection si-TXNDC9/si-NC. n= 4, *P<0.05. (**F**) The caspase3 activity of U87 and U251 cells was evaluated by the caspase3 activity kit. n= 6, **P<0.01. (**G**) The protein level of p53, Cleaved-caspase3, Cleaved-caspase8, and Cleaved-caspase9 were detected by western blot, Gapdh was indicated as a loading control. n= 6, *P<0.05.

For further study, we constructed siRNA (si-TXNDC9) for inhibiting the function of TXNDC9, si-NC (negative control) was described as control. The clone formation experiment showed si-TXNDC9 reduced the number of clones (Figure 2A). Then we discussed the effect of TXNDC9 on glioma cell migration and invasion. Wound healing assay and transwell showed that si-TXNDC9 significantly inhibited cell migration and invasion (Figure 2B, 2C). After transfection with si-TXNDC9/si-NC, the cell apoptosis rate was measured by flow cytometry; the results showed that the percent of apoptosis cell was significantly increased in the si-TXNDC9 group (Figure 2D). Then the cell cycle assay was performed. The knockdown of TXNDC9 blocked cells from the G0 phase to the S phase (Figure 2E). Next, we determined the Caspase3 activity with the Caspase 3 Activity Assay Kit. The loss function of TXNDC9 induced caspase3 activation (Figure 2F). In previous studies, p53 played an essential role in regulating tumor progression [16–18]. Then we evaluated the expression of p53 in cells after transfecting with si-TXNDC9/si-NC; Down-regulated of TXNDC9 induced expression of p53 increased, and the apoptosis-associated protein (Cleaved-caspase3, Cleaved-caspase8, and Cleaved-caspase9) (Figure 2G).

### Effects of TXNDC9 on cell autophagy in U87 and U251 cells

**Figure 3:**
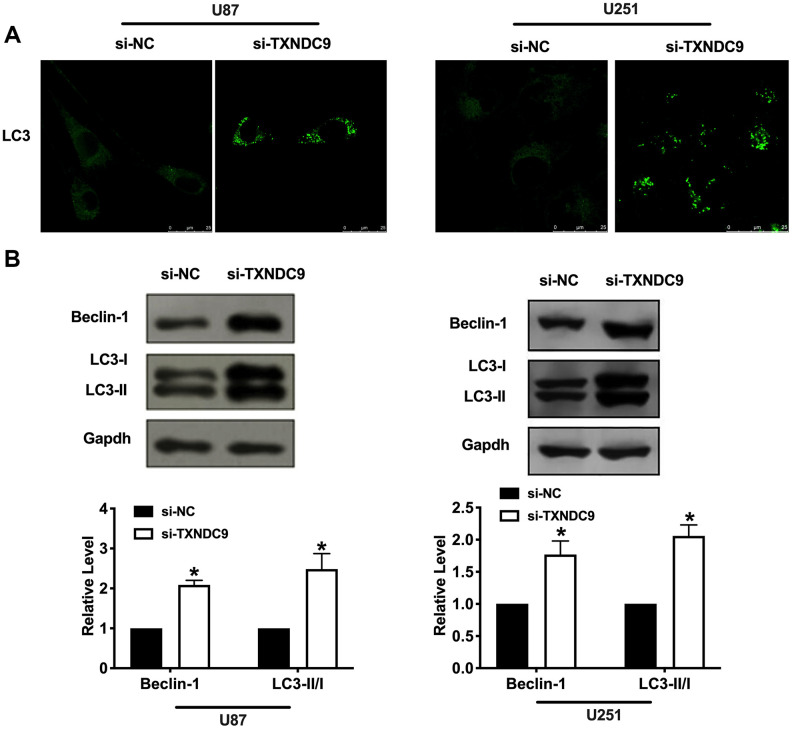
**Knockdown of TXNDC9 promoted autophagy in U87 cells.** (**A**) The level of GFP-LC3 and localization in U87 and U251 cells after transfecting with si-TXNDC9/si-NC. Representative immunofluorescence images were shown. (**B**) The protein level of Beclin-1 and LC3-I/II was detected in U87 and U251 cells, Gapdh was indicated as a loading control. n= 6, *P<0.05.

Autophagy degrades itself through the lysosome pathway, which is of considerable significance to the survival, development, balance, and differentiation of cells, and plays a protective role in the body. In the local hypoxic microenvironment of the tumor, it will promote the occurrence of autophagy. We constructed LC3 fused to green fluorescent protein (GFP-LC3) and transfected it into U87 and U251 cells. After transfection si-TXNDC9/si-NC, we found that knockdown of TXNDC9 induced the up-regulated of LC3, which showed induction of autophagy (Figure 3A). Beclin-1, LC3-I, and LC3-II were detected by western blot analysis, and the conversion of LC3 was demonstrated by LC3-II/LC3-I ratio. The results showed that si-TXNDC9 promoted the level of Beclin-1 and LC3-II/LC3-I (Figure 3B). In summary, si-TXNDC9 could induce autophagy in U87 and U251 cells.

### Differentiation of U87 and U251 cells by TXNDC9

**Figure 4:**
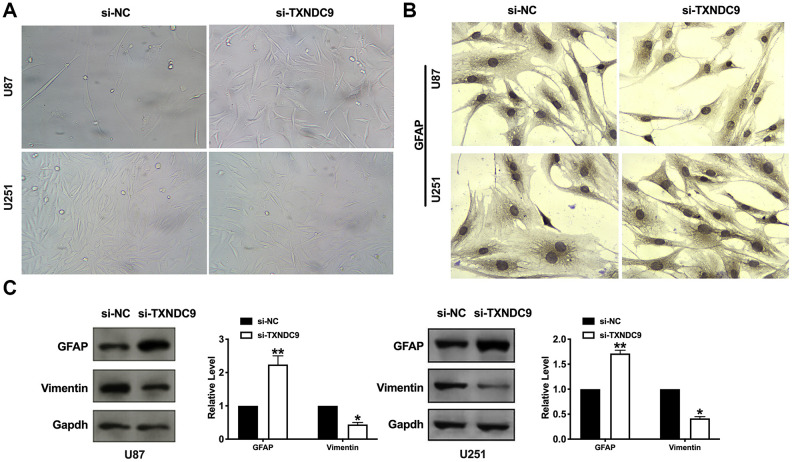
**Knockdown of TXNDC9 induced differentiation of glioma cells.** (**A**) U87 cell morphology was scanned after transfecting with si-TXNDC9/si-NC. (**B**) The immunocytochemical assay was performed for GFAP expression. (**C**). The protein level of vimentin and GFAP were measured in U87 and U251 cells, Gapdh was indicated as a loading control. n= 6, *P<0.05, **P<0.01.

Inducing tumor cells to differentiate into normal cells or nearly normal cells has become a hot spot in the research of antineoplastic drugs. In vitro studies have shown that tumor cells can differentiate under the induction of some preparations, some have a normal phenotype, and some have restored some functions of normal cells. U87 and U251 cells observed by microscope showed that knockdown of TXNDC9 induced a significant change in structural morphology. Compared with the si-NC group, the shape of U87 and U251 glioma cells were long fusiform, the processes increase and become longer, and differentiate obviously, similar to normal astrocytes. (Figure 4A). We also explored whether morphology changed was corrective with GFAP expression; the image showed that si-TXNDC9 induced morphology changed accompanied by up-regulating of GFAP (Figure 4B). Vimentin and GFAP were indicated as markers of early and late glial differentiation [19], western blot results revealed that knockdown of TXNDC9 inhibited the expression of vimentin and promoted the expression of GFAP (Figure 4C). Taken together, knockdown of TXNDC9 would facilitate the differentiation of U87 and U251 cells.

### TXNDC9 regulates procession of glioma cells via controlling p53

**Figure 5:**
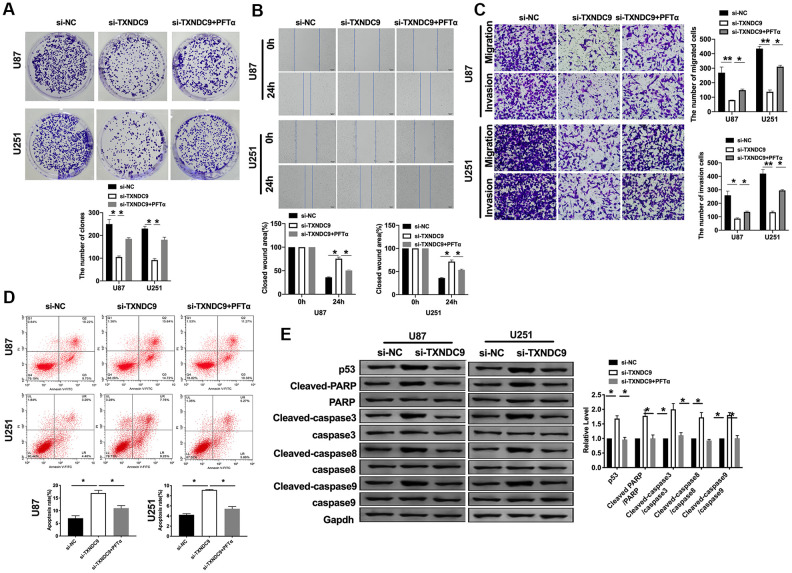
**TXNDC9 regulated glioma program via controlling p53.** (**A**) The colony formation assay. (**B**, **C**) Wound healing assay and transwell were performed for detecting the effect of p53 on migration and invasion. (**D**) The apoptosis rate of U87 and U251 cells was measured after si-TXNDC9/si-NC transfection and PFTα treatment. The histogram at the right is a statistical graph. n=6, *P<0.05. (**E**) The protein level of p53, Cleaved-caspase3, Cleaved-caspase8, and Cleaved-caspase9 were detected by western blot, Gapdh was indicated as a loading control. n= 6, *P<0.05.

Based on the above results, we explored the mechanism of TXNDC9 in U87 and U251 cells. P53 has been deeply studied in a variety of human tumors. Most researchers believed that p53 is closely related to tumor invasion and metastasis and, to a certain extent, associated with the prognosis of patients. In previous studies, we found that si-TXNDC9 would induce the expression of p53 (Figure 2G). The U87 and U251 cells were treated with 10 μM p53 inhibitor (PFTα) for 48 h. As Figure 5A shown, PFTα additional treatment prevented the inhibition of si-TXNDC9 on the colony formation. Wound healing and transwell assay results showed that PFTα would restore the ability of migration and invasion (Figure 5B, 5C). In Figure 5D, PFTα additional treatment decreased the apoptosis rate. Meanwhile, si-TXNDC9 induced the expression of apoptosis-related protein that was inhibited by PFTα treated (Figure 5E).

**Figure 6:**
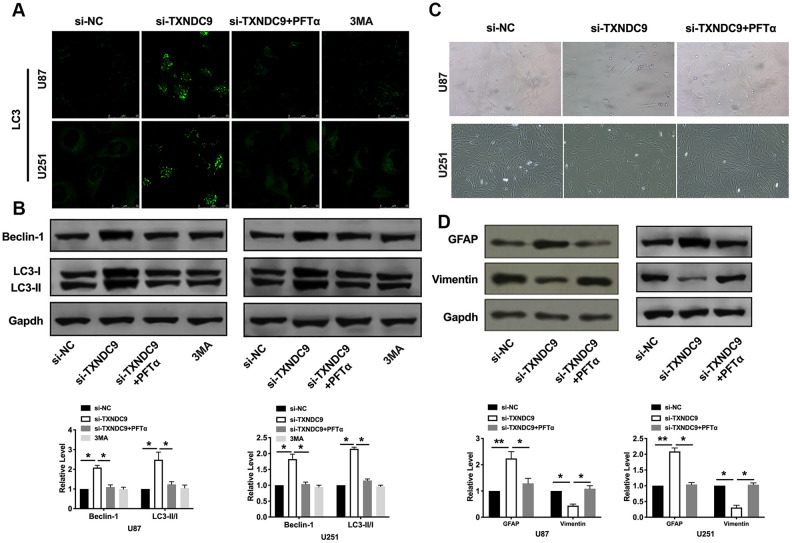
**TXNDC9 regulated glioma autophagy and differentiation via controlling p53.** (**A**) The level of LC3 and localization in U87 and U251 cells after si-TXNDC9/si-NC transfection, PFTα, and 3MA (5 mM) treatment. Representative immunofluorescence images were shown. (**B**) The protein level of Beclin-1 and LC3-I/II was detected in U87 and U251cells; Gapdh was indicated as a loading control. n= 6, *P<0.05. (**C**) U87 and U251 cell morphology were scanned after si-TXNDC9/si-NC transfection and PFTα treatment. (**D**) The protein level of vimentin and GFAP were measured in U87 and U251 cells, Gapdh was indicated as a loading control. n= 6, *P<0.05, **P<0.01.

PFTα also altered the fluorescence intensity of LC3 and decreased the level of Beclin-1 and the ratio of LC3- II/LC3-I (Figure 6A, 6B). Observed morphology through a microscope, PFTα reversed the change caused by si-TXNDC9 (Figure 6C). The level of vimentin and GFAP also showed that PFTα made cells in a state of low differentiation (Figure 6D). In conclusion, TXNDC9 involved in apoptosis, autophagy, and differentiation of glioma cells via regulating p53.

## DISCUSSION

In this study, we found the up-regulated of TXNDC9 in U87 and U251 glioma cells. Knockdown of TXNDC9 could prevent cell metastasis and induced apoptosis, which was indicated by increasing the level of apoptosis-associated proteins. Meanwhile, the Knockdown of TXNDC9 could induce autophagy, which was described by the up-regulated of Beclin-2 and the LC3-II/LC3-I ratio. As the morphological changed and increasing vimentin, GFAP, si-TXNDC9 induced differentiation of U87 and U251 cells. During this program, the activation of p53 played a key role.

In recent years, intracranial tumors account for an increasing proportion of nervous system diseases, of which glioma accounts for more than half. Patients with low-grade glioma still cannot avoid recurrence after treatment. Still, for patients with high-level glioma, it brings specific challenges to clinical workers and patients whether to treat and what kind of treatment to take [20, 21]. Even if surgery plus radiotherapy and chemotherapy are adopted, the prognosis is not optimistic in the later stage [22]. This is not only an excellent challenge for clinical workers and researchers at home and abroad but also a heavy burden for patients and their families [23]. But the research on glioma has not stopped.

Inducing tumor cell apoptosis and autophagy is a convenient means to inhibit tumor development [24, 25]. Tumor suppressor p53 family proteins widely regulate phagocytosis, apoptosis, cell cycle, metabolism, DNA repair, and so on. They all play an inhibitory role in the tumor. P53 is down-regulated or deleted in most human tumors. The regulation of p53 on autophagy is related to its spatial distribution and plays a dual role. P53 in the nucleus mainly inhibits mTOR and induces autophagy through AMPK and TSC1/2 pathways [26]. P53 also induces tumor suppressor gene PTEN to inhibit PI3K/AKT signal pathway, induce apoptosis, and inhibit cancer cell migration [27]. Polychlorinated biphenyl quinone induced signaling transition from autophagy to apoptosis is regulated via p53 in human hepatoma HepG2 cells [28].

At present, inducing tumor cell differentiation is an effective way of treatment, and some key issues regulating the signal pathway of tumor cell differentiation are gradually being clarified: the entanglement and dialogue between different signal pathways; the preventive and regulatory effects of various kinases on different stimuli in the signal pathway; and the dose-effect and the time-effect relationship between cell differentiation and signal kinase activation. It was reported that notch signaling regulates oral neoplasm cell differentiation [29]. Inhibition of the EGFR/AKT signaling pathway promotes ovarian cancer cell differentiation via regulating TSA [11]. Prostaglandin E2 promotes immune escape via the inhibition of natural killer cell differentiation [30]. In our study, we found that TXNDC9 can affect the differentiation of glioma cells.

TXNDC9 is a member of the thioredoxin family. It can bind to ATP and maintain the redox state in the cell. The TXNDC9 gene is located at 2q11.2. It consists of 17,374 base pairs. It encodes a protein with a molecular weight of 27 ku and contains 226 amino acid residues. The protein is distributed in the cytoplasm. It includes the N-terminal and acidic C-terminal of the helical structure. It gets its name because it contains the same domain as thioredoxin in the center. In recent years, studies have found that. Many members of the Thioredoxin family are mainly involved in the redox reaction of the body. It is highly expressed in many solid tumors such as liver cancer [14], colon cancer [31], breast cancer [32] and so on. Other experiments showed that TXNDC9 was up-regulated in some oxaliplatin-resistant strains. At present, there are few studies on the role of TXNDC9 protein in eukaryotic cells. The specific function of the protein is not known. The expression and function of TXNDC9 in glioma cells have not been reported.

In this study, for the first, the function of TXNDC9 was revealed in glioma cells. Knockdown of TXNDC induced apoptosis and autophagy of glioma cells and promoted differentiation through regulating p53.

## MATERIALS AND METHODS

### Clinical samples

The tumor samples were collected from 35 glioma patients at Peking University Shenzhen Hospital. The characteristics of the 35 cases of newly diagnosed glioma patients involved in the study cohort. The mean age was 51.5 years, with a range of 24 to 79. There were 21 (60%) males and 14 (40%) females. According to the WHO classifications in 2007, 7 (20%),13(37.1%), 10 (28.6%), and 5 (14.3%) of 35 glioma patients were classified as WHO grade I, II, III, and IV. The normal tissues were collected from paired adjacent tissues. All of the patients or their guardians provided written consent. This research has got the approval of the Medical Ethics Committee of Peking University Shenzhen Hospital, and this study is in line with the Declaration of Helsinki.

### Cell culture

The cell lines (NHA, LN18, U87, U118, T98, U251) were purchased from the Science Cell Laboratory. Cell lines were cultured in PRIM 1640 (Thermo-life, United States) with 10 % FBS (Thermo Fisher, USA) and 100 μL/mL penicillin and streptomycin (Beyotime, China) and placed at 37°C with 5% CO2. The cells were treated with 10 μM p53 inhibitor, Pifithrin-α (PFTα) (Selleck, USA)for 48 h and 5mM 3MA for 12 h.

### Western blot

Total protein was collected from cells with RIPA lysis Mix (Beyotime, China). Briefly, 60 μg protein extraction was loaded via SDS-PAGE and transferred onto nitrocellulose membranes (MILLIPORE, USA), then put them into a 5% blocking solution for 3 h. The membranes were incubated with primary antibodies at 4 °C for one night. After incubation with secondary antibodies, the membranes were scanned using an Odyssey, and data were analyzed with Odyssey software (LI-COR, USA). p53 (60283-2-Ig, 1:500), Cleaved-caspase3 25546-1-AP, 1:500), LC3I/II(14600-1-AP, 1:1000), Beclin-1 (11306-1-AP, 1:500), Vimentin (60330-1-Ig, 1:500) and GFAP (16825-1-AP, 1:500) were purchased from proteintech; Cleaved-caspase8 (WL0153,1:500), Cleaved-caspase9 (WL01838, 1:500) were purchased from Wanlei (Wanleibio, China), Gapdh (60004-1-Ig, 1:2000) was used as an internal control.

### Real time-PCR

Total RNA was isolated from glioma cells according to a standard protocol. And then, the purity and concentration of RNA were detected, and all the samples were converted into cDNA using reverse transcription kit. We used SYBR Green (Thermo Fisher Scientific) system to perform the qRT-PCR. Data were analyzed by GraphPad 7.

### Wound-healing assay

The wound-healing assay was carried out on U87 and U251 cells. 5 ×10^5^ cells were cultured in trans-well plates, and then the cells were gently scratched with a pipette tip. The fresh medium was changed. After columbamine treatment, the scratched spaces on the plate were evaluated by microscopy.

### Matrigel invasion assay

Cells in the logarithmic growth phase were adjusted to 2 × 10^5^ cells/well of medium (without serum) and plated 1μg/μl Matrigel into the upper chamber. The lower chamber was added with 500 μL of the medium, and then incubate the plate at 37°C for 48 h. Then the invading cells were visualized by the crystal violet and inverted microscope.

### Immunocytochemistry

2×10^5^ of U87 cells and U251 were cultured in 24-well plates. Then cells were fixed in paraformaldehyde (4%) for 0.5 h at room temperature. The endogenous peroxidase activity was abolished with H_2_O_2_ (3%) in methanol (10%)/PBS for 10 min, and 15 min for permeabilization with Triton X-100 (0.5%). After 1 h incubation with serum, it was added with primary mouse anti-GFAP antibody (16825-1-AP, 1:200) for 2 h. Cells were then incubated with diaminobenzidine substrate for 5 min and then finally counterstained with hematoxylin and mounted with glycerol (50%).

### Confocal imaging

Cells were seeded in 24-well plates and transfected with GFP-LC3 for U87 and U251 cells. 24 h after transfection, the cells were transfected with siRNA for an additional 48 h. Cells were fixed with 4% paraformaldehyde in PBS for 30 min at room temperature; the cells were mounted in anti-fading solution and stored at 4°C. The plates were examined under a laser microscope.

### Caspase3 activity assay

Caspase3 activity assay was performed by the Caspase 3 Activity Assay Kit (Beyotime, China). Absorb the cell culture medium and set aside. The adherent cells were digested with trypsin and collected into a spare cell culture medium. The cells were collected by centrifugation at 600g at 4 °C for 5 minutes, and the supernatant was carefully removed. At the same time, no cells were absorbed as far as possible, and PBS was washed once. After absorbing the supernatant as before, add the lysate according to the proportion of 100 microliter lysate for every 2 million cells, re-suspension precipitation, ice bath cracking for 15 minutes. Operate according to instructions. Take out the right amount of Ac-DEVD-pNA (2mM) and set aside on the ice bath. Add Ac-DEVD-pNA (2mM) and mix well note. Incubate for 120 minutes at 37 °C. A405 can be determined when the color change is obvious. The A405 of the sample deducts the A405 of the blank control, that is, the absorbance produced by the pNA catalyzed by caspase 3 in the sample.

### Cell apoptosis assay

The cells were counted about 5×105cells/mL. Then, 1 mL cells were centrifuged, 1000 rpm, 10 min, 4°C, and the medium was throw away. The cells were washed with PBS and dropped medium. The cells were resuspended and avoid light for 15 min, 200 μL Binding Buffer with 10 μL Annexin V-FITC, and 10 μL PI. Flow cytometry was used to measure the apoptosis rate within 1 h.

### Cell cycle assay

Cells were collected with 1ml trypsin for 2min, suspension the cell with 5ml PBS, centrifuge at 1000 RPM for 5 min at 4°C. 10ml PBS buffer was used to re-washed and dropping medium, Then the cells were fixed with 70% ethanol overnight. The next day, the cell medium was filtered with a 300-mesh sieve, centrifuged at 1000 RPM at 4°C for 5min, and the supernatant was discarded. The cells were avoided light and fixed with 1ml PI solution and stated at 4°C for 30 min. Flow cytometer was used to evaluate the cell cycle.

### Statistical analysis

All values are expressed as the mean ± SEM. Statistical significances were measured by Student’s t-test and ANOVA. A two-tailed value of P < 0.05 was indicated as a statistically significant difference. Data statistics were used the GraphPad 7.0.
