# Correction for: Knockdown of TXNDC9 induces apoptosis and autophagy in glioma and mediates cell differentiation by p53 activation

**DOI:** 10.18632/aging.205623

**Published:** 2024-02-15

**Authors:** Tingting Zheng, Keke Chen, Xue Zhang, Huanhuan Feng, Yu Shi, Li Liu, Jun Zhang, Yun Chen

**Affiliations:** 1 Shenzhen Key Laboratory for Drug Addiction and Medication Safety, Department of Ultrasound, Peking University Shenzhen Hospital, Shenzhen Peking University, The Hong Kong University of Science and Technology Medical Center, Shenzhen, Guangdong Province, China; 2 Clinical College of Shenzhen Hospital, Peking University, Anhui Medical University, Shenzhen, Guangdong Province, China; 3 School of Materials Science and Engineering, Harbin Institute of Technology Shenzhen, Shenzhen, Guangdong Province, China; 4 Queensland Micro- and Nanotechnology Centre, Griffith University, Brisbane, Australia

**Keywords:** glioma, TXNDC9, apoptosis, autophagy, differentiation

**This article has been corrected:** The authors found that two images overlap in **Figure 5C**, which depicts transwell assays assessing the effects of TXNDC9 on migration and invasion by glioma cells. The panel illustrating invasion by U87 cells treated with the p53 inhibitor PFTα after si-TXNDC9 transfection (si-TXNDC9+PFTα) was replaced with the appropriate image from the original set of experiments. This alteration does not affect the results or the conclusion drawn from this work.

The corrected **Figure 5** is presented below.

**Figure 5:**
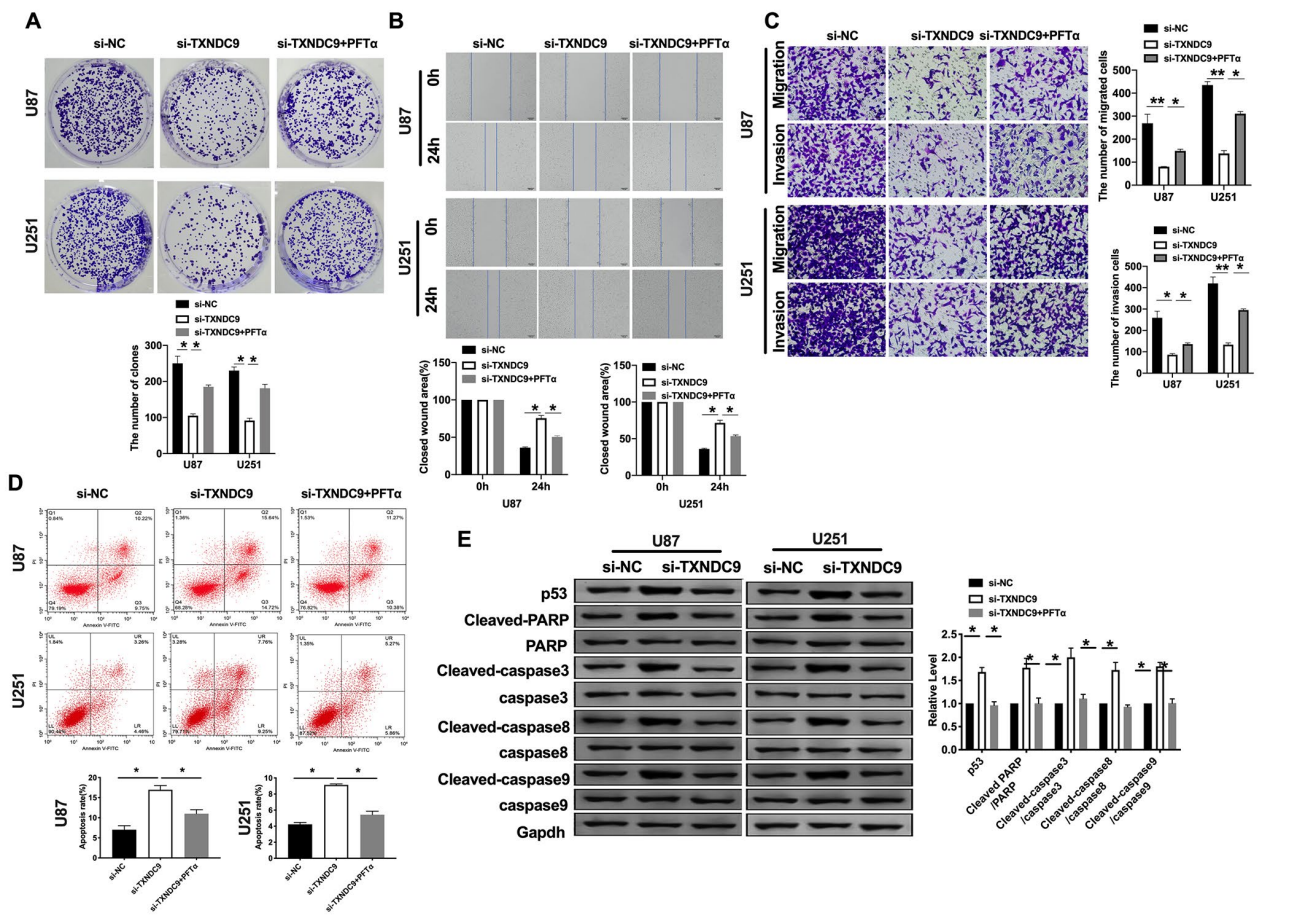
**TXNDC9 regulated glioma program via controlling p53.** (**A**) The colony formation assay. (**B**, **C**) Wound healing assay and transwell were performed for detecting the effect of p53 on migration and invasion. (**D**) The apoptosis rate of U87 and U251 cells was measured after si-TXNDC9/si-NC transfection and PFTα treatment. The histogram at the right is a statistical graph. n=6, *P<0.05. (**E**) The protein level of p53, Cleaved-caspase3, Cleaved-caspase8, and Cleaved-caspase9 were detected by western blot, Gapdh was indicated as a loading control. n= 6, *P<0.05.

